# From dementia mindsets to emotions and behaviors: Predicting person-centered care in care professionals

**DOI:** 10.1177/14713012221083392

**Published:** 2022-05-05

**Authors:** Lena K Kunz, Susanne Scheibe, Barbara Wisse, Kathrin Boerner, Claudia Zemlin

**Affiliations:** Department of Psychology, 403520 University of Groningen, Groningen, The Netherlands; Department of Psychology, 403520 University of Groningen, Groningen, The Netherlands; Department of Gerontology, 14708 University of Massachusetts Boston, Boston, MA, USA; Department of Human Medicine, 163484 University of Witten/Herdecke, Witten, Germany; Dementia Care Unit, Vitanas GmbH & Co. KGaA, Germany

**Keywords:** Caregiving–formal, education and training, long-term care, nursing homes, quality of care, workforce issues

## Abstract

**Background and Objective:**

High-quality care standards for dementia care are increasingly based on person-centered care principles. To better understand facilitating factors of person-centered care this research focuses on individual characteristics of care professionals. Applying mindset theory to dementia care, we examined dementia mindsets (viewing dementia symptoms as either malleable or fixed) in care professionals. We tested whether there is a positive relationship between a malleable dementia mindset and person-centered care as well as a negative relationship between a fixed dementia mindset and person-centered care. Moreover, we examined whether care professionals’ emotional responses in care situations help explain associations between dementia mindsets and person-centered care.

**Research Design and Method:**

In two cross-sectional studies, care professionals of long-term care facilities (total *N* = 370) completed a measure of dementia mindsets and reported their emotional and behavioral responses to five care scenarios. Regression and mediation analyses were performed.

**Findings:**

The tested hypotheses were partially supported. A fixed dementia mindset predicted reported person-centered care negatively, while a malleable dementia mindset did not. Mediation analyses suggest that reduced negative emotions may underlie the association between a malleable mindset and reported person-centered care, while reduced positive emotions in care situations may underlie the association of a fixed mindset and reported person-centered care. Study 2 partially replicated these findings. A fixed mindset and positive emotional responses were the most robust predictors of reported person-centered care.

**Discussion and Implications:**

This study extends knowledge on facilitators (positive emotional responses to care situations) and barriers (fixed dementia mindset) to person-centered care in care professionals working with persons with dementia. We discuss how dementia mindsets and emotional responses to care situations may be a fruitful target for trainings for care professionals.

Perceptions of what constitutes high-quality care for persons with dementia have shifted over the past decade. While early research and practice were dominated by a biomedical approach characterized by the focus on medical treatments of dementia, the field has moved towards individualized care and the person-centered philosophy (Fazio et al., 2018). Rather than treating dementia and related behaviors primarily with pharmacological products, this latter approach aims to focus on the person behind the diagnosis, including personal history, needs, and (dis)likes during care provision. As such, person-centered care is rooted in authentic social interactions and focuses on meeting psychological needs (e.g., comfort, occupation, identity), supporting opportunities for meaningful activities (Kitwood, 1997), and fostering a holistic view of the person. Person-centered care as such contributes to a reduction in dementia-related behaviors/reactions (e.g., anxiety and depression) and thereby can enhance day-to-day quality of life of persons with dementia (Kim & Park, 2017).

Given positive effects of person-centered care on the care and support of persons with dementia, it is important to understand factors that facilitate person-centered care. Research suggests that next to contextual factors (e.g., organizational culture, Røen et al., 2018), personal factors in care professionals contribute to the provision of person-centered care (Hunter et al., 2016). Among these personal factors, prior research has mainly focused on the role of knowledge about dementia, acquired skills, and beliefs about personhood – defined as the extent to which persons are seen in a holistic way, including their distinctiveness, personal links to the past, present and future, and need for autonomy. This research suggests that care providers with stronger beliefs in personhood provide person-centered care more frequently (Hunter et al., 2013). Yet, beliefs about personhood do not include the care professional’s assumptions about the changeability of dementia symptoms and associated well-being through their own and others’ actions or adjustments of the care environment, both physically and socially. Such assumptions may be highly relevant as person-centered care requires constantly reevaluating care practices and altering malfunctioning strategies (Fazio et al., 2018).

This additional nuance in how care professionals view dementia symptoms is captured by the concept of dementia mindsets that can be either malleable or fixed. Dementia mindsets are defined as the belief that expressions and progression of dementia symptoms, and how they affect persons’ quality of life, can (in case of a malleable mindset) or cannot (in case of a fixed mindset) be influenced by the social or physical environment (Kunz et al., 2020). Dementia mindsets are important in the care context, as they predict job-related well-being in care professionals. For example, a malleable dementia mindset predicted lower levels of disengagement, a facet of burnout, and higher sense of competence (Kunz et al., 2020). However, care professionals’ behavioral consequences of dementia mindsets in care situations have not yet been explored.

To address this gap, we examine the relationship between dementia mindsets–the implicit theories that care professionals hold about dementia, and person-centered care behaviors in two samples of care professionals. Based on mindset theory, which holds that people view human attributes as either malleable (adaptable or changeable) or fixed (inflexible or constant; Dweck & Leggett, 1988), we predict that care professionals with a malleable dementia mindset are more likely to report person-centered care provision, as their actions are driven by the belief that quality of life of persons with dementia can be enhanced by an altered physical and social environment. Contrary, we expect those with a fixed dementia mindset to be less likely to report person-centered care provision. We further argue that this may occur because a malleable mindset fosters positive emotional reactions (e.g., feeling delighted or calm) and diminishes negative emotional reactions (e.g., annoyed or depressed) to care situations while the reverse pattern of associations may be expected for a fixed mindset. The underlying mechanisms are based on the broaden-and-build theory of positive emotions (Fredrickson, 1998), which suggests that the experience of positive emotions provides a gateway to novel and creative ideas, while the experience of negative emotions inhibit such processes and foster disengagement. These positive and negative emotional reactions, in turn, may further predict the provision of person-centered care. Taken together, we aim to contribute to a better understanding of personal factors that predict who is motivated and able to provide person-centered care to persons with dementia in professional settings.

## Effects of person-centered care

Person-centered care has developed into the gold standard to support persons with dementia (Brooker, 2003). Centered on the person, not the diagnosis or symptoms, it is characterized by sincere interpersonal interactions and caregivers’ respect for the care recipients’ individuality focused on psychological needs (Kitwood, 1997), rather than on deficits associated with dementia. For instance, an 83-year-old former athlete and now resident at a long-term care facility, pacing around the ward and showing restlessness during meals, receives person-centered care if care staff find suitable solutions considering his professional history and associated needs to adjust daily routines.

Based on person-centered care, several programs to initiate cultural change in nursing homes (e.g., Eden Alternative, Green Houses) have been developed (Li & Porock, 2014). Today, the philosophical foundation of person-centered care serves as the basis of the 2018 Alzheimer’s Association Dementia Care Practice Recommendations. These include (1) knowing the person living with dementia, (2) recognizing and accepting the person’s reality, (3) identifying ongoing support opportunities for meaningful engagement, (4) building and nurturing authentic, caring relationships, (5) creating and maintaining a supportive community for individuals, family, and staff, and (6) evaluating care practices regularly and making appropriate changes (Fazio et al., 2018).

Evaluation studies suggest that person-centered care practices are effective, contributing to lower levels of expressions of unmet needs, increased positive affect in persons with dementia, and reductions in pharmacological treatments (Li & Porock, 2014), and facilitating general quality of life of persons with dementia through more interpersonal interactions (Yasuda & Sakakibara, 2017). Moreover, person-centered care has beneficial effects on care professionals (Brownie & Nancarrow, 2013), such as enhanced job satisfaction, reduced stress and burnout (Barbosa et al., 2015).

## Predictors of person-centered care: The role of caregivers

Positive effects of person-centered care on persons with dementia and care professionals underscore the importance of nursing education programs that foster this approach and contribute to its facilitation in practice. However, which individual factors predict whether care professionals adopt person-centered care? A commonly used framework differentiates knowledge, abilities, skills, and other characteristics (KSAO) relevant to the nursing context (McCormack & McCance, 2006). Research indicates that performing person-centered care is difficult without knowledge (K) about dementia (Bamford et al., 2019). Moreover, skill-based training programs (S) for staff resulted in more person-centered care (Blake et al., 2019). Beneficial skills may range from communication skills (Morris et al., 2020) such as validation techniques (Feil, 2014) to performing Dementia Care Mapping (DCM, Kitwood & Bredin, 1997). Other characteristics (O) found to facilitate person-centered care include beliefs about personhood that promote empathy and respect for persons with dementia (Hunter et al., 2016), and increased empowerment of registered nurses in care delivery (Marriott-Statham et al., 2018). Additionally, a review on mental health nursing demonstrated that anger among care professionals predicted negative behaviors towards clients, jeopardizing quality of care (Jalil & Dickens, 2018). This highlights the relevance of emotional experiences of care professionals as important personal characteristic in context of care provision and support.

In sum, care professionals’ knowledge, skills, attitudes, beliefs, and other personal attributes are essential to facilitate person-centered care. Yet, a focus on care professionals’ belief that dementia symptoms and reduced quality of life can be alleviated through care professional-initiated changes in the external world needs further research attention. Our study seeks to contribute to these research efforts with a closer examination of individual differences in dementia mindsets.

## Individual differences: dementia mindsets

Recently, the concept of dementia mindsets was introduced and a scale to measure it was developed (Kunz et al., 2020). This concept originates from mindset theory (Dweck & Leggett, 1988) and captures the idea that care professionals adopt fixed or malleable dementia mindsets to varying degrees. With a strong fixed mindset, dementia is viewed as a neurodegenerative disease characterized by inevitable decline in physical, cognitive, and social abilities, and reduced quality of life. When people have a strong malleable mindset, they also view dementia as a neurodegenerative disease or disability, but one that does not necessarily eradicate quality of life of persons with dementia. This entails that symptoms of dementia can be influenced by adjustments of environmental circumstances and social interactions to better meet individual needs. In a series of studies with care professionals in dementia care, the two mindsets were found to form two independent factors that were negatively related to each other (Kunz et al., 2020). This suggests that care professionals can adopt both mindsets simultaneously, with one being more pronounced than the other. Findings also showed that care professionals with a stronger malleable dementia mindset evaluated their own competence of providing care to persons with dementia more positive and felt that their work was more fulfilling. While dementia mindsets in care professionals seem to be relevant for well-being at work, no research has yet examined their impact on behavioral outcomes, specifically on person-centered care.

We predict that a malleable dementia mindset will be positively related to person-centered care, while a fixed mindset will be negatively related to person-centered care. Indeed, person-centered care requires care professionals’ acceptance of behaviors that challenge as “a consequence of an unmet need with the focus of the intervention being to meet the need” (James, 2011, p. 105). These interventions need to undergo a constant reevaluating of care plans and require a sensitive and mindful eye for unsuccessful or non-fitting strategies (Fazio et al., 2018). Previous studies on general mindsets found the malleable mindset to result in greater mastery and less helplessness in face of setbacks (Lou & Li, 2017). Thus, individuals with a malleable dementia mindset are more likely to adapt to difficulties compared to those with a fixed dementia mindset who are more likely to show helpless behavior. Moreover, we predict that malleable and fixed dementia mindsets have unique relationships with the experience of certain emotions, which in turn predict the likelihood that person-centered care is actually provided.

## The mediating role of emotions in care professionals

Several studies show that mindsets can impact the person’s own emotional state. For instance, malleable mindsets contribute to affective well-being (Bernecker et al., 2017). Likewise, a malleable view about intergroup conflict is associated with the experience of more positive emotions (hope), while a fixed mindset is associated with the experience of increased negative emotions (anxiety; Rattan & Georgeac, 2017). Further, people who pursue future goals experience more positive emotions and less negative emotions when they have adopted a malleable rather than a fixed mindset (Burnette et al., 2013). Accordingly, we expect the malleable mindset to relate positively to positive emotional reactions to care situations and negatively to negative emotional reactions. We expect the reverse pattern of associations for a fixed mindset.

Persons’ feelings are also known to affect how they perform their tasks and interact with others at work (Jalil & Dickens, 2018); both are crucial aspects of respectful interactions with persons with dementia in person-centered care (Kitwood, 1997). The broaden-and-build theory of positive emotions (Frederickson, 1998) addresses underlying mechanisms that explain why increased positive emotions and decreased negative emotions experienced by those with a malleable mindset (vice versa for those with a fixed mindset) may facilitate person-centered care. The theory suggests that positive emotions allow more novel and creative ideas, and a stronger focus on personal relationships—both of which are relevant to person-centered care. Novel and creative solutions are particularly relevant for non-pharmacological and non-invasive interventions in daily interactions with persons with dementia, as they require reactions tailored to individual needs. They often require creative and unconventional approaches such as providing a spot to retreat when personal interactions or noises are overwhelming and persons with dementia might react aggressively.

Further, the broaden-and-build theory suggests that negative emotions inhibit thought–action repertoires. Negative emotional reactions to the behavior of persons with dementia in care situations might block creative ideas on how to resolve or react to such situations, leading to disengagement or avoidance. Therefore, positive emotional reactions to care situations (feeling calm) may allow access to creative solutions, personal development, and stronger social connections. In contrast, negative emotional reactions (feeling annoyed) might restrict access to these types of creative solutions. Thus, we expect that emotional reactions to care situations not only matter in the provision of person-centered care but also explain the relationship between dementia mindsets and person-centered care.

### The present research

Based on the reviewed literature we hypothesize the following.

Hypothesis 1 Dementia mindsets predict person-centered care: (a) A malleable dementia mindset predicts person-centered care positively while (b) a fixed mindset predicts person-centered care negatively.

Hypothesis 2 Emotional responses to care situations predict person-centered care: (a) Positive emotional responses predict higher levels of person-centered care, while (b) negative emotional responses predict lower levels of person-centered care.

Hypothesis 3 Emotional responses in care situations mediate the relationship between a malleable dementia mindsets and person-centered care: (a) A malleable mindset indirectly predicts more person-centered care through enhanced positive emotional responses, while (b) a malleable mindset indirectly predicts more person-centered care through reduced negative emotional responses.

Hypothesis 4 Emotional responses in care situations mediate the relationship between a fixed dementia mindsets and person-centered care: (a) A fixed dementia mindset indirectly predicts less person-centered care through reduced positive emotional responses, while (b) a fixed dementia mindset indirectly predicts less person-centered care through increased negative emotional responses.

We conducted two studies in collaboration with a large nursing home agency in Germany with a specialized department for facilitating person-centered care through trainings, consulting, and a (re)certification process. Study 1 included a heterogeneous group of care professionals (e.g., direct care workers, occupational therapists) of multiple nursing homes. Study 2 included a more select group responsible for recreational and occupational tasks (e.g., enhancing activity-based interventions), also from multiple facilities. Both studies were approved by the Ethical Committee of the University of Groningen.

### Study 1: Method

#### Respondents and procedure

A group of 778^1^ care professionals of a German care provider were invited to participate; of those, 221 completed the survey (response rate 28.4%). We excluded 17 responses with more than 10% missing values, and 31 responses that lacked relevant demographic information, leaving us with a final sample of 173 care professionals. Sample characteristics are provided in Table 1. Top/middle management and the agency’s worker council, composed of staff delegates, approved data collection. Paper-and-pencil questionnaires, accompanied by a return envelope, were distributed among employees. We assured interested care professionals of anonymity and confidentiality and informed them they could withdraw from the study at any time without penalty.

**Table 1.** Demographic characteristics of care professionals in Study 1 and Study 2.

Variables	Study 1	Study 2
Age		
Younger than 29	19.7%	5.9%
30–39	19.1%	16.5%
40–49	27.2%	21.8%
Older than 50	34.1%	55.8%
Gender		
Female	86.6%	91.7%
Male	13.4%	8.3%
Profession		
Certified nurse	51.4%	1.6%
Direct care worker	27.7%	2.7%
Therapeutic and recreational staff	-	88.3%
Others	19.8%	7.4%
Frequency of contact with persons with dementia		
Several times per hour	62.6%	70.9%
Several times a day	36.3%	29.1%
About once a day	1.2%	-
Less often than once a day	-	-

*Note.* The category Others included professions such as Therapists, Case Manager, Care Coordinators. Managing Staff and Nurses in Training.

### Measures

#### Dementia mindsets

Care professionals completed the Dementia Mindset Scale (see Supplementary Materials, Appendix A), a 12-item measure (Kunz et al., 2020). Six items measure a malleable dementia mindset (“Despite their gradual decline in attention span, persons with dementia are still able to engage in meaningful tasks when opportunities are provided”), while the other six items measure a fixed dementia mindset (“There is nothing one can do about the increasing disorientation in persons with dementia”). Items were rated on a 5-point scale ranging from 1 (strongly disagree) to 5 (strongly agree).

### Reported provision of person-centered care

To measure the tendency to provide person-centered care, we used five vignettes (Dementia Care Style Questionnaire: Brooker et al., 1998; German translation; Seidl & Walter, 2012). The measure is designed as a situational judgment test, also referred to as low-fidelity simulation; this type of measure generally predicts actual job behavior well (Lievens & Motowidlo, 2016). Respondents were presented with scenarios like: “Mrs H. was happy the entire day. Suddenly, without an apparent reason, she bursts into tears,” and provided with four response options (one of them representing person-centered care – see Supplementary Materials, Appendix B, for the complete measure). They were asked which option best described their likely course of action. In the example, the person-centered behavior option is “I go to Mrs H and sit beside her. I try to empathize with her and see if she can bring herself to explain why she feels so sad.” We counted the number of times individuals selected the person-centered care approach across the five scenarios (range from 0 to 5). We treated person-centered care as a continuous variable, based on significance of the Kolmogorov–Smirnov test (*p* < .01), which suggests that the distribution of person-centered care is significantly different from a Poisson distribution (as is common for the distribution of count variables).

### Situation-specific emotional response

In each scenario, care professionals also indicated how they would feel in the described situation. Two positive emotions (delighted and calm) and two negative emotions (annoyed and depressed) were presented and rated on a five-point scale ranging from 1 (not at all) to 5 (very strongly). Two average scores were computed for positive and negative emotional responses. Positive and negative emotional responses were moderately negatively correlated (r = −0.36, *p* < .01).

### Control variables

We added chronological age and tenure as covariates as prior studies report age-related differences in affective reactivity to work events (Scheibe et al., 2019), and a positive relationship between care professionals’ work experience and person-centered care in context of dementia caregiving (Zimmermann et al., 2014).

## Study 1: findings

### Descriptives

Table 2 displays descriptive statistics, correlations and Cronbach’s α of study variables. Mindsets show a negative intercorrelation (r = −0.27, *p* < .01), suggesting that care professionals can have both mindsets simultaneously, while one of the two is more dominant. Care professionals reported higher scores on the malleable dementia mindset (M = 4.38, SD = .52) than on the fixed mindset (M = 2.48, SD = .79; t(173) = 23.49, *p* < .01). Reports of negative emotions were relatively low (M = 1.38, SD = .38) as compared to those on positive emotions (M = 3.25, SD = .56; t(173) = 31.43, *p* < .01). Age was negatively related to positive emotional responses (r = −0.16, *p* = .04) and tenure was positively related to reported provision of person-centered care (r = .18, *p* = .02).

**Table 2.** Descriptives, correlations, and internal consistencies of variables in study 1.

	M	SD	Min	Max	1	2	3	4	5	6	7
1 Age^a^	2.78	1.20	−	−	(−)						
2 Tenure (years)	9.31	8.81	<1	40	−.34***	(−)					
3 Malleable dementia mindset	4.38	0.52	2.33	5.00	.04	.07	(.73)				
4 Fixed dementia mindset	2.48	0.80	1.00	4.83	−.11	−.07	−.27***	(.77)			
5 Person-centered care	3.42	1.18	0	5.00	−.08	.18*	.16*	−.23***	(−)		
6 Positive emotions	3.25	0.56	1.88	4.80	−.16*	.04	.09	−.22***	.48***	(.78)	
7 Negative emotions	1.38	0.38	1.00	2.90	−.02	−.08	−.26***	.24***	−.47***	−.37***	(.77)

*Note.* Positive and Negative Emotions refer to Situation-Specific Emotional Responses. Internal Consistencies (Cronbach’s alpha) of the measures is displayed in parenthesis on the diagonal of the correlation table.

^a^Age was assessed in decades (0 =19 or younger; 6 = 60 or older). Frequencies are reported in Table 1.

*p = .05, ** *p* = .01. ***, *p* < .01.

### Predicting reported provision of person-centered care

We tested our hypotheses by running mediation analyses using the PROCESS macro (Version 3.4, Hayes, 2017; Model 4). The model included the two dementia mindsets as predictors, positive and negative emotional responses as mediators, and reported person-centered care as outcome, controlling for age and tenure. As PROCESS only allows testing indirect effects for one predictor at a time, we ran the model twice, switching the position of the two mindsets (one as predictor and the other as covariate).

**Figure 1.**
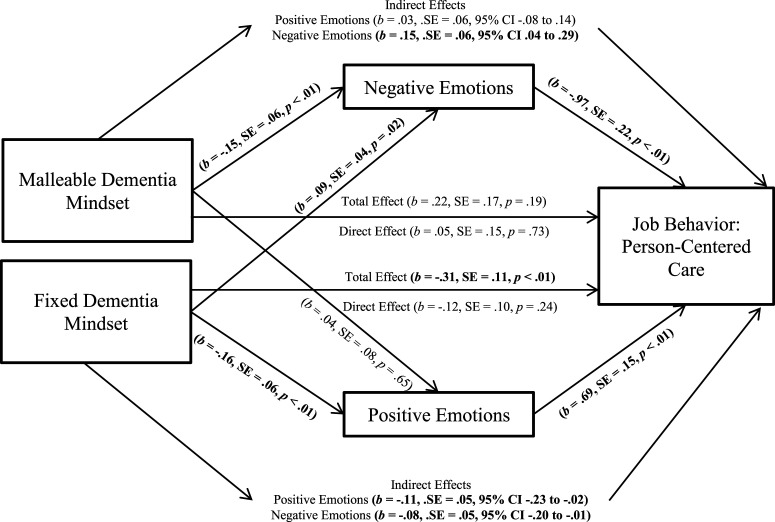
Mediation Model of Study 1. Note. *N* = 173. Mediation model of situation-specific emotional responses on the relationship between dementia mindsets and job behavior (person-centered care) in Study 1. Controlling for age, tenure, and (either malleable or fixed) dementia mindset. The total effect of dementia mindsets on person-centered care = indirect effect (dementia mindsets on person-centered care via emotional responses) + direct effect (dementia mindsets on person-centered care).

The fixed dementia mindset was negatively related to person-centered care, while a malleable dementia mindset was unrelated^2^ (Figure 1). Further, the malleable dementia mindset was unrelated to positive emotional responses but negatively related to negative emotional responses. The fixed dementia mindset was negatively related to positive emotional responses, but positively related to negative emotional responses. Both positive and negative emotional responses predicted person-centered care: Positive emotional responses predicted more person-centered care and negative emotional responses predicted less person-centered care. The indirect effect of a malleable dementia mindset on person-centered care through negative emotional responses was significant; yet the indirect effect through positive emotional responses failed to reach significance. The indirect effects of a fixed dementia mindset on person-centered care through positive emotional responses and negative emotional responses were significant. There was no direct effect of a malleable dementia mindset on person-centered care, yet the direct effect of a fixed dementia mindset on person-centered care was significant.

In sum (Table 3), findings largely supported our hypotheses. A malleable dementia mindset positively predicted reported person-centered care due to reduced negative emotions (in line with H3b), while positive emotions did not explain this relationship (contrary to H3a). Further, a fixed dementia mindset was negatively related to the provision of reported person-centered care (supporting H1b), through positive and negative emotional responses (supporting H4a and H4b). Additionally, both emotional responses predicted reported person-centered care (in line with H2a and H2b), such that positive emotional responses positively predicted reported person-centered care, while negative emotional responses negatively predicted reported person-centered care.

**Table 3.** Overview of hypotheses and findings of Study 1 and Study 2.

Hypotheses		Findings	
		Study 1	Study 2
H1a	A malleable dementia mindset predicts person-centered care positively	Not supported	Not supported
H1b	A fixed dementia mindset predicts person-centered care negatively	Supported	Supported
H2a	Positive emotional responses positively predict person-centered care	Supported	Supported
H2b	Negative emotional responses negatively predict person-centered care	Supported	Not supported
H3a	A malleable mindset indirectly predicts more person-centered care through enhanced positive emotional responses	Not supported	Not supported
H3b	A malleable mindset indirectly predicts more person-centered care through reduced negative emotional responses	Supported	Not supported
H4a	A fixed dementia mindset indirectly predicts less person-centered care through reduced positive emotional responses	Supported	Not supported
H4b	A fixed dementia mindset indirectly predicts less person-centered care through increased negative emotional responses	Supported	Not supported

### Study 2: method

#### Respondents and procedure

A group of 264^3^ care professionals of the same German care provider as in Study 1 participated in Study 2. To increase data-quality we deleted the data of 62 respondents with more than 10% missing values, and five respondents who had little contact with persons with dementia daily, leaving us with a final sample of 197 care professionals. Sample characteristics are provided in Table 1. We followed the same procedure as in Study 1, only this time care professionals were handed the study material during an annual training on communication skills in the context of dementia care. Although the training was mandatory, participation in the study was not. We explicitly informed training participants that study participation was completely voluntary. To avoid any social demand effects, respondents could decide whether to complete the questionnaire on site or at home. A return envelope was provided to anonymously send the questionnaire back.

### Measures

Measures used in Study 2 were similar to Study 1. Exceptions are described below.

### Dementia mindsets

We used a short version of the Dementia Mindset Scale with four items assessing a malleable dementia mindset and four items assessing a fixed dementia mindset

### Reported provision of person-centered care

We used the same scenario measure as in Study 1. Results of the Kolmogorov–Smirnov test suggests that the distribution of person-centered care was significantly different from a Poisson distribution (*p* = .03), again enabling us to treat it as a continuous variable.

### Situation-Specific Emotional Responses

In Study 1, negative emotional responses showed a floor effect, probably because both negative emotion items (annoyed and depressed) were considered socially undesirable in care situations. In this study, we therefore included a wider range of emotional responses: four positive (cheerful, delighted, comfortable, content) and four negative emotions (uncomfortable, puzzled, miserable, discouraged). Two average scores were computed, one for positive and one for negative emotional responses.

### Study 2: findings

#### Descriptives

Table 4 displays descriptive statistics, correlations, and Cronbach’s α of study variables. Contrary to Study 1, malleable and fixed dementia mindsets were unrelated in this sample (r = −0.02, *p* = .82). Negative and positive emotional responses were relatively low (M = 1.86, SD = .51; M = 2.33, SD = .48, respectively). In general, participants reported higher scores on the malleable dementia mindset (M = 4.09, SD = .52) than on the fixed dementia mindset (M = 2.52, SD = .84).

**Table 4.** Descriptives, correlations, and internal consistencies of variables in Study 2.

	M	SD	Min	Max	1	2	3	4	5	6	7
1 Age^a^	3.40	1.11	0	5	(−)						
2 Tenure (years)	23.83	15.31	0	47.00	.57***	(−)					
3 Malleable mindset	3.75	0.77	1.25	5.00	−.08	.04	(.70)				
4 Fixed mindset	2.52	0.84	1.00	4.75	.03	.12	−.04	(.69)			
5 Person-centered care	2.97	1.26	0	5.00	−.09	−.14	.06	−.33***	(−)		
6 Positive emotions	2.33	0.48	1.15	4.00	−.14	−.10	−.04	<−.01	.15*	(.86)	
7 Negative emotions	1.86	0.51	1.00	3.65	−.07	−.09	−.28***	.09	−.12	.20***	(.84)

*Note.* Positive and Negative Emotions refer to Situation-Specific Emotional Responses. Internal Consistencies (Cronbach’s alpha) of the measures are displayed in parenthesis on the diagonal.

^a^Age was assessed in decades (0 = 19 or younger; 6 = 60 or older). Frequencies are reported in Table 1.

*p = .05, ** *p* = .01. ***, *p* < .01.

### Predicting reported provision of person-centered care

**Figure 2.**
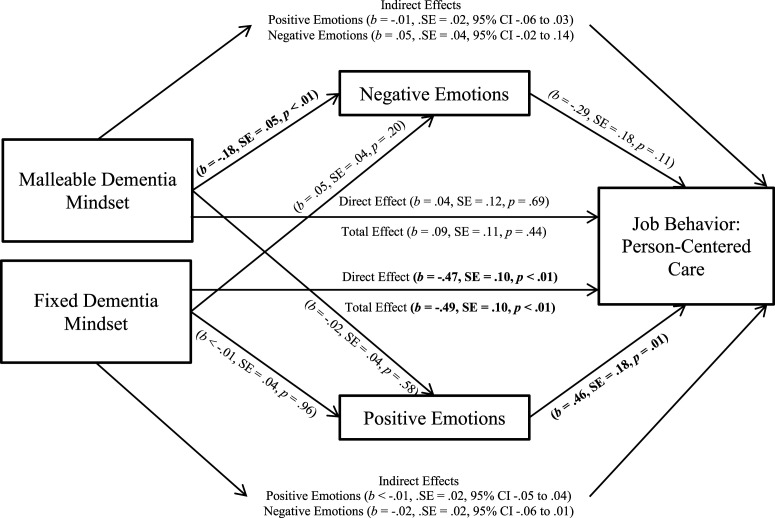
Mediation Model of Study 2. Notes. *N* = 197. Mediation model of situation-specific emotional responses on the relationship between dementia mindsets and job behavior (person-centered care) in Study 2. Controlling for age, tenure and (either malleable or fixed) dementia mindset. Controlling for age, tenure and (either malleable or fixed) dementia mindset. The total effect of dementia mindsets on person-centered care = indirect effect (dementia mindsets on person-centered care via emotional responses) + direct effect (dementia mindsets on person-centered care).

We adopted the same analytical approach as in Study 1, using the PROCESS macro to estimate direct and indirect effects of dementia mindsets on person-centered care through emotional responses. As shown in Figure 2 and replicating findings of Study 1, the fixed dementia mindset negatively predicted person-centered care, while the malleable mindset was unrelated to person-centered care. Further, findings suggest that the malleable dementia mindset negatively predicted negative emotional responses, while a fixed dementia mindset did not. Positive emotional responses predicted person-centered care positively, while negative emotional responses were unrelated to person-centered care.

The indirect effects of a malleable dementia mindset on person-centered care through negative emotional responses and positive emotional responses were not significant; neither were the indirect effects of a fixed dementia mindset on person-centered care through positive or negative emotional responses. The direct effect of a malleable dementia mindset was not significant, yet the direct effect of a fixed dementia mindset on person-centered care was again significant.

In sum (Table 3), the findings of Study 2 provide partial support for the overall model. The malleable dementia mindset predicted reported person-centered care neither directly (failing to support H1a) nor indirectly through emotional responses (failing to support H3). In line with findings of Study 1, fixed dementia mindset predicted reported person-centered care negatively (supporting H1b); but this could not be explained by positive or negative emotional responses (failing to support H4). Notably, positive emotional responses were related to reported person-centered care (supporting H2a), while negative emotional responses were unrelated to reported person-centered care (failing to support H2b).

## Discussion

Two cross-sectional studies with dementia care professionals examined the role of dementia mindsets in reported person-centered care, and the role of emotional responses to care events as a potential mechanism underlying this relationship. Findings of both studies support the relevance of care professionals’ dementia mindsets and emotional responses to daily care situations for self-reported person-centered care. A fixed mindset and positive emotional responses were the most robust predictors. Specifically, holding a fixed dementia mindset–thus, believing that dementia symptoms are unchangeable and that nothing can be done to prevent that dementia diminishes quality of life–was associated with a lower likelihood of person-centered care, highlighting the restrictive role cognitive rigidity can have on individuals’ behavioral responses. On the other hand, positive emotional responses were associated with increased person-centered care reporting; thus, care professionals who reported feeling calm and other positive emotions in care situations also reported more authentic and respectful behavioral reactions. Further, findings showed that emotional responses, such as feeling delighted or annoyed, may account for the relationship between dementia mindsets and person-centered care, providing first insights into the underlying mechanisms. Below, we also discuss potential reasons for why not all findings of Study 1 were replicated in Study 2.

Our replicated findings emphasize the usefulness of considering dementia mindsets of care professionals as a relevant, yet so far neglected individual-level predictor of person-centered care. Prior research outlined the role of knowledge (K), skills (S), abilities (A), and other factors (O), such as beliefs in personhood (Hunter et al., 2013). Dementia mindsets represent another factor in the O domain, capturing beliefs in the changeability of dementia and therewith a unique nuance in personal factors. As such, dementia mindsets seem to determine the extent to which care professionals exert effort in arranging the care environment such that the needs of a person with dementia are met. Consequently, educational programs for care professionals can benefit from integrating dementia mindsets to facilitate high quality care for persons with dementia.

Our findings showed that specifically a fixed rather than a malleable dementia mindset was predictive of self-reported work behavior. Although perceiving dementia symptoms as malleable and flexible predicts caregiver engagement levels and feelings of competence (Kunz et al., 2020), it may not directly affect behavioral responses. Possibly, a malleable dementia mindset by itself is not enough to provide effective and well-designed person-centered care—perhaps it only predicts behavior if care professionals also have the required skills, such as empathy (Brownie & Nancarrow, 2013), knowledge and opportunities to adjust behavior toward person-centered care. Moreover, care professionals with a fixed dementia mindset might be unwilling or unable to search for various options to react to a specific care situation, due to cognitive inflexibility, the inability to adapt and effectively react to new situations (Cañas et al., 2003). A fixed dementia mindset implies that care professionals believe their behavior to be without an impact and might therefore react according to well-known patterns, rather than trying new and different strategies. Prior research highlights the importance of cognitive flexibility as one function of cognitive control in a formal care setting that can be challenging, but is necessary for practicing person-centered care (Austrom et al., 2016).

In line with the broaden-and-built theory (Frederickson, 1998), our findings consistently support the hypothesis that positive emotional responses facilitate individualized care solutions. Positive emotions may give the care professional access to a wider array of solutions and increase openness towards multiple options (Haager et al., 2014). Also, positive emotions may a stronger focus on the personal relation to the person with dementia, which is a hallmark of person-centered care and essential for the dynamic interactive process between care professionals and persons with dementia.

Negative emotional responses were negatively related to person-centered care in Study 1 but not in Study 2. This may be due to sample differences as care professionals in Study 2 were considerably older than those in Study 1. Previous research has found that older adults are better at regulating their emotions and more likely focus on positive information inherent in a given situation, such as a difficult care situation (Charles & Carstensen, 2014). Thus, negative feelings may have less of an impact on the behavior of older care professionals. Further, the difference in educational level and background of care professionals between the two studies might have played a role, as care professionals in Study 1 obtained a more time intensive training with a greater focus on person-centered care compared to care professionals in Study 2. Additionally, higher educational backgrounds might also contribute to higher social and emotional competencies at work (Gilar-Corbi et al., 2018) that allow a differentiated view on one’s own emotional responses to daily care situations. Alternatively, the difference in findings across the two studies may be the result of the measurement differences, which either may not have captured the most typical emotional responses in care situations (Study 1), or did not capture high intensity emotions (Study 2). The mediating role of emotional responses might specifically be relevant in case of strong emotional responses to care situations.

### Limitations and future research

First, both samples were recruited from the same agency, possibly resulting in reduced variance of responses. Future research should consider recruiting care professionals from different long-term care agencies, diverse cultural backgrounds and taking additional measures to address non-response. Second, power analyses showed that both studies, especially in regard to the role of emotional responses, require a larger sample size to detect small effects, which should be considered when interpreting the findings. Third, due to lack of a validated measure for emotions in care situations, our measure of emotional responses was self-developed. Future studies might include other measures of emotional responses (including physiological or observational ones) and/or control for social desirability. Emotional responses could be assessed throughout the workday to better map care professionals’ emotions and how these drive their behaviors. Fourth, person-centered care was measured through a situational judgment test that could be hampered by limits to care professionals’ willingness and ability to self-reflect on their behavior in care situations. Observational measures such as the Dementia Care Mapping (Kitwood & Bredin, 1997) allow objectively coding care behavior of professionals.

Finally, although we expected dementia mindsets to be associated with particular emotions and behaviors in care situations, we could not determine causality, due the cross-sectional design. Experiences during care situations, such as failing to fulfill a resident’s needs, may trigger the impression that dementia symptoms are unchangeable, despite the best intentions. Future research may adopt longitudinal designs, mapping care professionals’ experiences and behaviors over time, and how these interplay with changes in dementia mindsets. Alternatively, researchers may test whether changing dementia mindsets through tailored interventions lead to changes in emotions and behavior in care situations over time. Indeed, prior research on mindsets in various domains indicates that mindset interventions possess the power to manipulate mindsets (Dweck & Yeager, 2019), opening up the possibility to conduct experimental research and make causal inferences possible.

## Conclusion

Our research draws attention to the relevance of care professionals’ dementia mindsets and emotional responses when working with persons with dementia. Findings on both dementia mindsets and emotional responses to care situations as predictors of reported person-centered care provision may provide a new angle for the development of trainings and interventions focused on fostering person-centered care. Such training programs and interventions may concentrate on fostering person-centered care by shaping care professionals’ views about dementia and learning effective emotion regulation strategies. Our study suggests that it may be particularly useful to break down fixed mindsets and increase the tendency to respond with positive emotions in care professionals.

## Supplemental Material

sj-pdf-1-dem-10.1177_14713012221083392 – Supplemental Material for From dementia mindsets to emotions and behaviors: Predicting person-centered care in care professionals Click here for additional data file. Supplemental Material, sj-pdf-1-dem-10.1177_14713012221083392 for From dementia mindsets to emotions and behaviors: Predicting person-centered care in care professionals by Lena K Kunz, Susanne Scheibe, Barbara Wisse, Kathrin Boerner and Claudia Zemlin in Dementia

## Notes

1 This is 36.59% of the total population of employees in relevant professions at the time of data collection (*N* = 2126). For Hypothesis 1 and 2, we used G*Power (Erdfelder, Faul, & Buchner, 1996) to calculate the a posteriori achieved power given our sample size. Assuming a one-sided alpha level of .05, our achieved power ranged between .37 to detect a small effect (r = .10) and .99 to detect a moderate effect (r = .30). For Hypotheses 3 and 4, we followed Fritz and MacKinnon’s (2007) estimated sample size requirements for indirect effects estimated through the bootstrap method. To achieve a power of .80, the required sample size ranges between 78 (assuming moderate effects for both the a and b paths) and 558 (assuming small effects for both the a and b paths). Thus, our achieved sample size was sufficient to detect moderate effects but suboptimal for detecting small effects.

2 Based on a hierarchical regression analysis, regressing a malleable dementia mindset on reported provision of person-centered care, findings suggest a significant positive effect. This is a robust finding when controlling for age and tenure excluding a fixed dementia mindset.

3 This is 96.35% of the total population of employees in relevant professions at the time of data collection (*N* = 274). As in Study 1, for Hypothesis 1 and 2 we used G*Power (Erdfelder, Faul, & Buchner, 1996) to calculate the achieved power. Assuming a one-sided alpha level of .05, our achieved power ranged between .41 to detect a small effect (r = .10) and .99 to detect a moderate effect (r = .30). For Hypotheses 3 and 4, the required sample size ranges again between 78 and 558 (see footnote 2). As for Study 1, this indicates that our achieved sample size was sufficient to detect moderate effects but suboptimal for detecting small effects.
