# Supplementary material for: From dementia mindsets to emotions and behaviors: Predicting person-centered care in care professionals
Source: Dementia (London). 2022 May 5;21(5):1618–35. doi: 10.1177/14713012221083392 (PMC9234781; doi:10.1177/14713012221083392)
Supplement: sj-pdf-1-dem-10.1177_14713012221083392 – Supplemental Material for From dementia mindsets to emotions and behaviors: Predicting person-centered care in care professionals [file sj-pdf-1-dem-10.1177_14713012221083392.pdf]

Appendices

Appendix A: Dementia Mindset Scale

Appendix B: Dementia Care Style Questionnaire

Appendix A

Dementia Mindset Scale – English Version

(Kunz, Scheibe, Wisse, Boerner, & Zemlin, 2020)

Views about Dementia

Instructions: The following statements capture different views of dementia. Please use the following scale to indicate your personal views of dementia. Please respond honestly and spontaneously.

(1 = *strongly disagree*, 2 = *somewhat disagree,* 3 = *neither agree nor disagree,* 4 = *somewhat agree*, 5 = *strongly agree)*

1. There is nothing one can do about the increasing disorientation in persons with dementia.
2. There is nothing one can do when persons with dementia become less able to engage in daily activities due to their declining attention span.
3. One cannot change the fact that persons with dementia are often less satisfied with their life because they can't express themselves as they used to.
4. Environmental adjustments will not change the progression of dementia.
5. No matter how much family caregivers and care professionals try, the progression of dementia symptoms cannot be slowed down.
6. Family and care professionals cannot change that persons with dementia are often insecure because they have a hard time doing things by themselves.
7. Despite their gradual decline in attention span, persons with dementia are still able to engage in meaningful tasks when opportunities are provided.
8. Engagement in daily activities enhances the sense of competence in persons with dementia, despite their declining ability to concentrate.
9. Some communication difficulties that are common in persons with dementia can be compensated for by caring and understanding interactions.
10. If persons with dementia are supported in their continuous adaptation to their disease, the increasing memory loss does not necessarily have to lead to emotional insecurities.
11. Conversations with persons with dementia can be meaningful despite verbal impairments, if family or care professionals take the time to engage in the conversation.
12. Being tolerant and understanding toward persons with dementia enables them to feel comfortable and to be themselves.

Fixed Dementia Mindset: Items # 1, 2, 3, 4, 5 and 6; Malleable Dementia Mindset: Items # 7, 8, 9, 10, 11 and 12.

Appendix B

Dementia Care Style Questionnaire

(Cited in Brooker et al., 1998; Adapted from German translation; Seidl & Walter, 2012)

Instructions: The following statements are about encounters with persons with dementia. Please indicate how you would react in the described scenario. There are no right or wrong answers!

Scenario 1: Mrs. D spends a large part of her day pacing up and down the living area. When she greets someone, she stops and gives them a kiss on the cheek.

What is your reaction? (Please choose an answer)

a) Considering this is an indication that the frontal lobes of the cortex are affected, I try to distract her from kissing anyone.
b) Considering this to be a sign of her affection, I try to encourage others to go with Mrs. D.
c) I tell her that this practice is foreign to others and try to explain the reasons why she does this.
d) I ignore her and the kissing. Once she regains her normal composure, go to her.

Scenario 2: Mrs. H. was happy the entire day. All of a sudden, without an apparent reason, she bursts into tears.

What is your reaction? (Please choose an answer)

a) It is most likely a catastrophic reaction. I will note the circumstances and time of the incident and include it in a report.
b) I go to Mrs. H and sit beside her. I try to empathize with her and see if she can bring herself to explain why she feels so sad.
c) People with Dementia forget quickly what evokes their emotions. Often, they experience sudden changes in mood. In light of this, I try to distract her and cheer her up.
d) I ignore the emotional outburst and only resume our conversation once she has regained her composure.

Scenario 3: You arrive on your ward after having a bad week. You are tired and still have a lot to do. Mr. S looks at you and says, “You look like you don’t feel too well today, my dear!”

What is your reaction? (Please choose an answer)

a) I tell him that I do not feel very well today. I thank him for noticing.
b) I notice that Mr. S is much less egocentric than usual. I ask myself if there have been changes to his medication. Other than that, I ignore the question.
c) I tell him that I have got a cold and that he does not need to worry about me.
d) I remind him that he should not be concerned about me, because it is my conviction that one should not burden patients with one’s own worries.

Scenario 4: Every afternoon Mr. S, a resident with Dementia, attempts to leave the living area. He says he needs to visit the post office in order to retrieve his pension check.

What is your reaction? (Please choose an answer)

a) One needs to make sure he has something interesting to do in the afternoons. For example, he should be given the opportunity to spend his money, which might give him a sense of security and trust.
b) This behavior is common for persons with Dementia. In order to calm his fears, one should tell him that the matter will be taken care of and the money will be collected for him.
c) One should pay attention to the exact time a day this happens. The right medication at the correct time could help.
d) One should tell him that he is in a Care Home/ Hospital and there is no reason why he needs to retrieve his pension check.

Scenario 5: Everyone is watching TV together, when Mrs. M gets up and starts singing an old song. She does so rather loud and off key.

What is your reaction? (Please choose an answer)

a) I try to distract her with a calming activity or try to direct her to another room.
b) I applaud and try to start a conversation about that song.
c) I ignore her behavior and try to sing a song together with the whole group, and include Mrs. M.
d) I tell her with a slight undertone of humor that she has an awful singing voice and ask her to stop singing.

Dementia Care Styles

P = Person-Centered Care Approach
B = Behavioral Approach
M = Medical Approach
N = Normalizing Approach

Answers	Scenario 1	Scenario 2	Scenario 3	Scenario 4	Scenario 5
a)	M	M	P	P	N
b)	P	P	M	B	P
c)	N	N	N	M	B
d)	B	B	B	N	M
